# Emerging Diagnostic Potential of Tumor-derived Exosomes

**DOI:** 10.7150/jca.59391

**Published:** 2021-06-16

**Authors:** Ruhua Luo, Mengmeng Liu, Qian Yang, Huijuan Cheng, Huimin Yang, Minhui Li, Xue Bai, Yue Wang, Honghua Zhang, Shuling Wang, Tian Xie, Qingchang Tian

**Affiliations:** 1 College of Pharmacy, School of Medicine, Hangzhou Normal University, Hangzhou, Zhejiang 311121, China.; 2 Key Laboratory of Elemene Class Anti-Cancer Chinese Medicines; Engineering Laboratory of Development and Application of Traditional Chinese Medicines; Collaborative Innovation Center of Traditional Chinese Medicines of Zhejiang Province, Hangzhou Normal University, Hangzhou, Zhejiang 311121, China.

**Keywords:** exosomes, tumor-derived exosomes, biomarkers, cancer detection

## Abstract

Exosomes carry genetic information originating from their parental cells, raising their possibility as novel noninvasive biomarkers for cancer. Tumor-derived exosomes (TEXs) have a variety of endogenous cargos that reflect the pathophysiology status and information of tumor cells. TEXs are increasingly being recognized as potential biomarkers for cancer diagnosis prognosis, and monitoring. It is important to develop a variety of sensitive methods, including probes and biomaterials to isolate exosomes. A variety of approaches for detecting exosomes have been established. By combining exosome DNA and RNA sequencing tools, exosome proteomics analysis and immunoassay technology, it is expected that exosomes will gain widespread use in the diagnosis and treatment of cancer.

## Introduction

Exosomes are small extracellular vehicles (EVs) ranging from ~30 to ~200 nm diameter that were released by almost all types of cells, including tumor cells, presenting pathophysiological roles and clinical value. Exosomes participate in mediating cell-to-cell communication, or multiple processes of tumor development, presenting pathophysiological roles and clinical value. They are nanosized membrane-bound nanovesicles that carry genetic information originating from their parental cells, raising their possibility as novel noninvasive biomarkers for cancer 1. Tumor-related exosomes have various contents that reflect pathophysiological changes in parental cells, whose information could be important in cancer diagnosis. Their presence in physiological fluids can be used to reveal important clinical information regarding these tissues 2. Exosomes have been widely reported in tumor research. Tumor-related exosomes are involved in the establishment or transformation of tumor microenvironments, advancing tumor angiogenesis and invasion, as well as mediating immune inhibition and escape 3. Exosomes with CD63 and caveolin-1 in plasma can be utilized as non-invasive markers of melanoma 4. Circulating exosomes with overexpressed glypican 1 (GPC1) in the serum of patients closely correlate with pancreatic cancer, which could be used as a highly specific biomarker for pancreatic cancer 5. Exosomes are involved in multiple processes of tumor genesis and development, including promoting angiogenesis 6, differentiation and infiltration 7, regulating immunity, and mediating drug resistance 8. Therefore, exosomes can be used as a potential biomarker for clinical applications and but exosomes-measuring technologies need to be standardized.

## Biological features of exosomes

**Figure 1:**
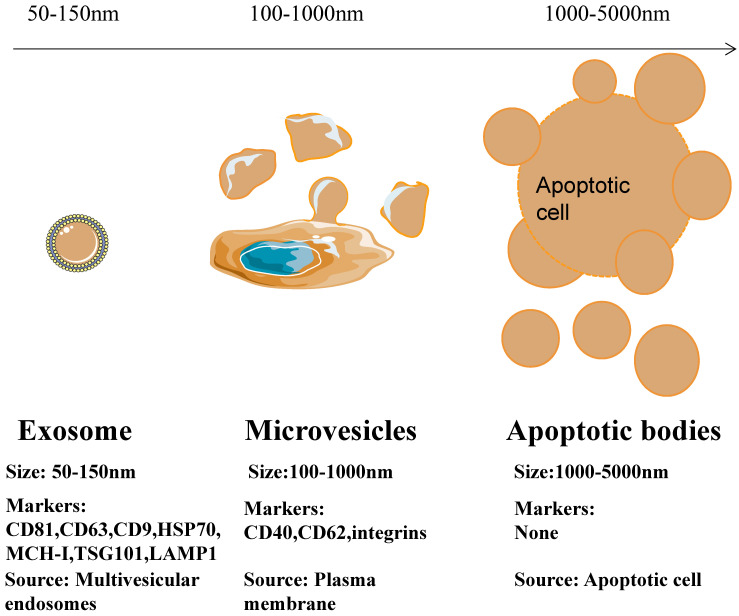
Differences in size, markers and origin of exosomes, microvesicles and apoptotic bodies.

**Figure 2:**
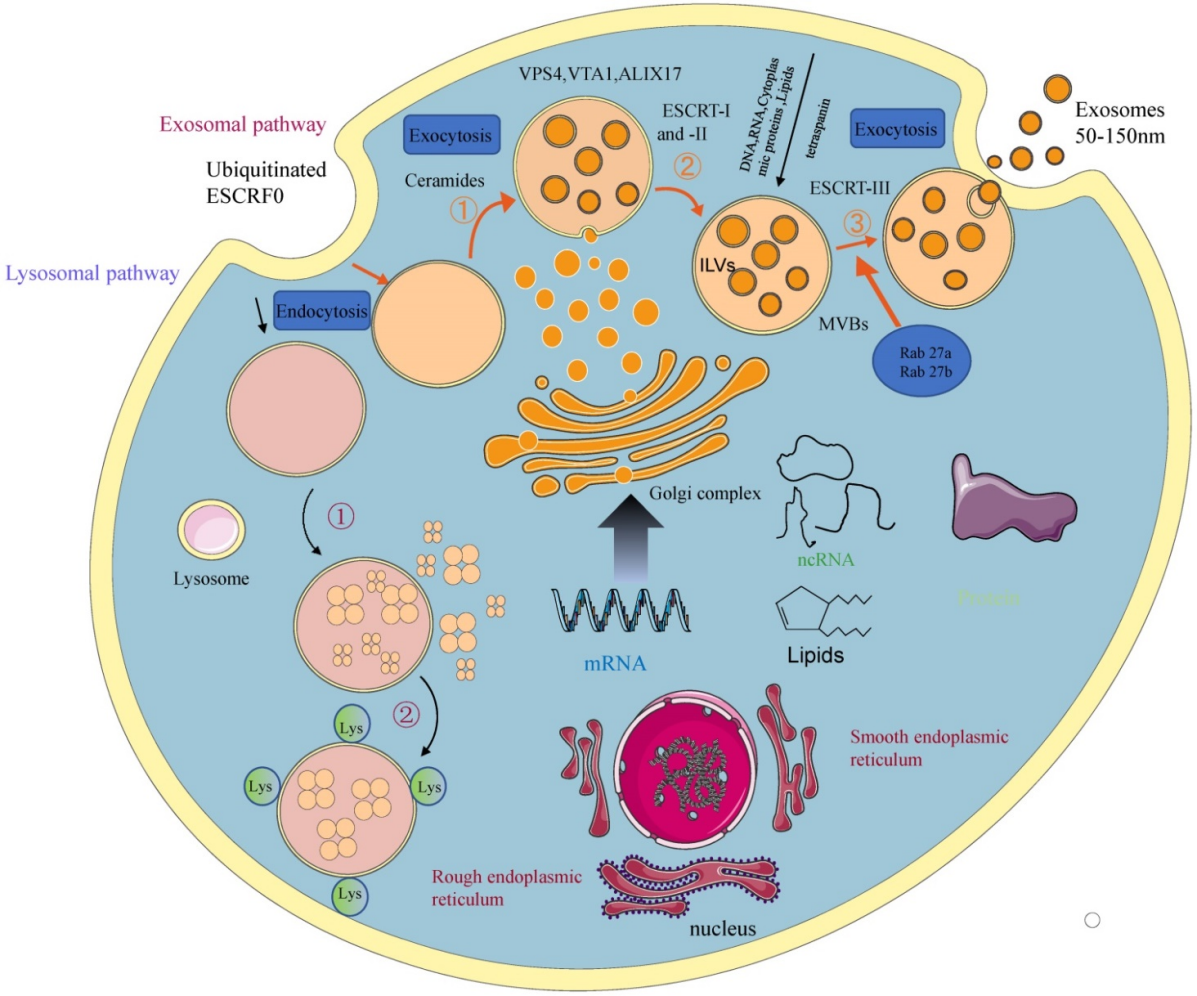
A schematic presentation of exosomal biogenesis. An early endosome is formed from the plasma membrane through the endocytic pathway. With the involvement of the Golgi apparatus, they mature into late endosomes and MVBs. The invagination from MVBs forms the ILVs. The process of exosomal biogenesis is dependent on either the ESCRT or independent of ESCRT mechanisms. Eventually, depending on the function and content of MVB, MVB fuses with the cell membrane and is released into the extracellular space as exosomes or fuse with lysosomes for degradation.

Depending on their biogenesis and generation pathways, EVs can be broadly classified into three subpopulations; exosomes, microvesicles, and apoptotic bodies 9 (Figure 1). Compared to microvesicles and apoptotic bodies, exosomes have a different biogenesis machinery 10. Exosomes are generated through a complex process (Figure 2). They are a class of cellular secreted nanovesicles that form multivesicular bodies (MVBs), which contain numerous intraluminal vesicles (ILVs).

Exosomes, as messengers of intercellular information transmission, are involved in the transfer of materials and information between cells by combining fusion or endocytosis 11. Its outer layer is the phospholipid bilayer similar to the cell membrane and related proteins such as membrane transport and fusion. Its interior contains functional biomolecules such as proteins and nucleic acids 12. Non-coding small RNA molecules (ncRNAs) are important in cell proliferation, differentiation and apoptosis 13.

## Potential of Exosomes in Tumor Diagnosis

**Figure 3:**
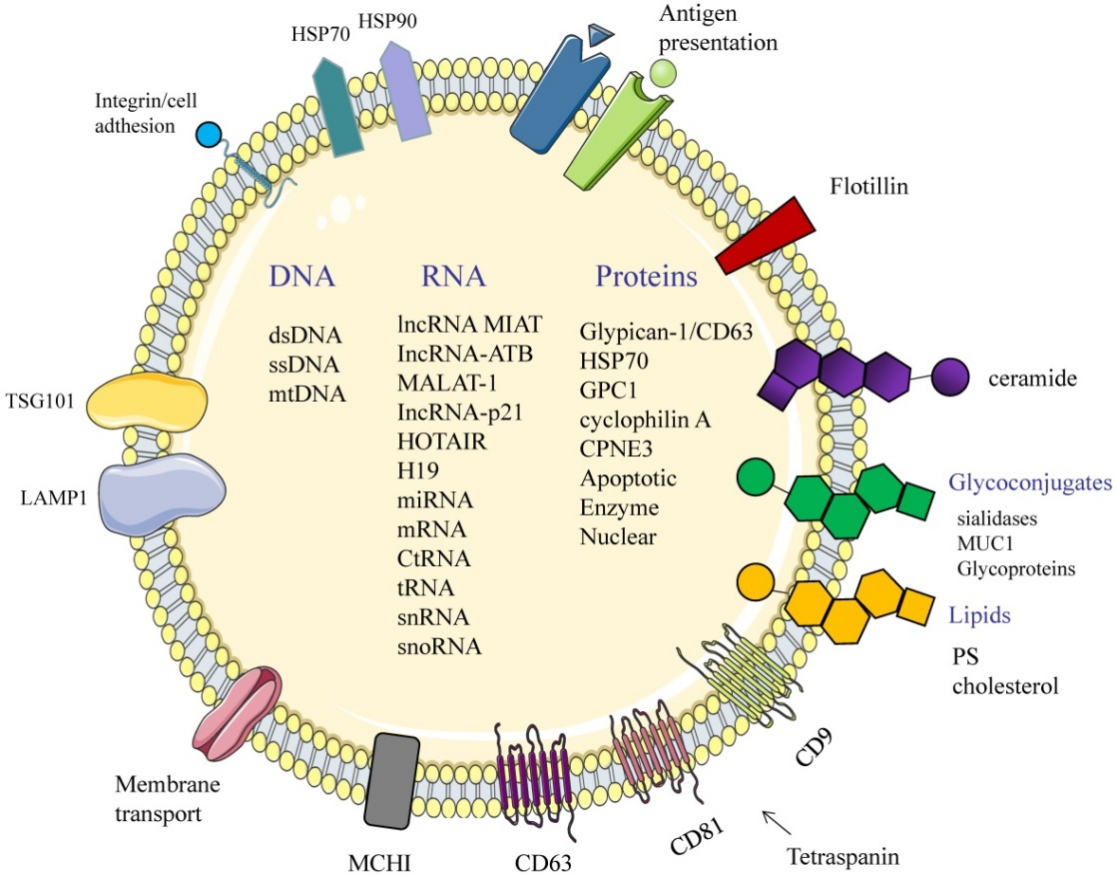
Structure and composition of exosomes. Exosomes consist of an aqueous core and a phospholipid bilayer. They carry important biomolecules such as proteins, DNA, and RNA, while the exosome membrane is rich in a variety of specific proteins, such as: Tetraspanins (CD9, CD63, CD81); major histocompatibility complexes I and II (MHC I, II); TSG101; HSP70, 90 (heat shock protein 70,90); LAMP1; Surface receptor; Ceramide; Glycoconjugates.

Early diagnosis and efficient therapy for patients are the best options for enhancing their survival outcomes. However, efficient diagnosis and therapy is inhibited by the lack of biomarkers that are highly specific, stable and noninvasive 14. As cancer biomarkers, the significance of exosomes in tumor diagnosis has been highlighted 15. Studies have documented that exosomes are important in intercellular communication and molecular exchange of constitutive lipids 16, characteristic proteins 17, functional messenger RNAs (mRNAs) and microRNAs (miRNAs) (Figure 3, Table 1) 18. TEXs carry a variety of endogenous cargos that reflect the pathophysiology status and information of tumor cells. Generally, cancer cells secrete more exosomes than their non-malignant counterparts. TEXs can be separated from body fluids to provide information about the tumor microenvironment. Accordingly, TEXs are increasingly being recognized as potential biomarkers for cancer diagnosis 19, prognosis, and monitoring 20.

### RNA

In recent years, liquid biopsies have been widely used in the early diagnosis of tumors, especially in the analysis of tumor-related substances extracted from body fluids of patients, such as miRNAs 59, exosomes 60 and circulating tumor cells (CTCs) 61. Exosomes contain various functional nucleic acids, some of which (such as miRNA, IncRNA and CircRNA) are promising options for cancer diagnosis. Studies have documented that exosomes play crucial roles in cancer onset, progression and metastasis regulating the stability of target mRNAs or by inhibiting translation 62. Although miRNAs have been well characterized in exosomes, a lack of specific expression and their small amounts limit their use as biomarkers 63. Recently, there are abundant data indicating that exosomal miRNAs play an vital role in cancer biology and may be potential diagnostic for cancer. A study indicated that there was a significant difference in the levels of serum exosomal microRNAs in patients with hepatocellular carcinoma (HCC) compared with patients with chronic hepatitis B (CHB) or liver cirrhosis (LC) 64. However, studies have shown that exosomal length non-coding RNA (IncRNA) has a significant therapeutic potential, including disease diagnosis 65. It has also been documented that TEXs IncRNA GAS5 is a potential diagnostic marker for early non-small cell carcinoma 66. Moreover, breast cancer preoperatively diagnosed as ductal carcinoma *in situ* (DCIS) patients are likely to develop invasive ductal carcinoma (IDC). miR-223-3p enhances breast cancer cell invasion, and its exosomes may be used as minimally invasive biomarkers for the diagnosis of IDC patients by biopsy 67. In addition, miR-375 68, miR-21 69 from urinary exosomes in both urine and blood have been identified to be specific exosomal miRNAs that can be used to distinguish between prostate cancer (PCa) patients from healthy individuals 70. Interestingly, studies used miRNA sequencing to investigate the exosomal miRNA profiles of patients with Papillary thyroid cancer (PTC). This method combined small RNA sequencing from numerous samples and RT-qPCR validation. A total of 41 significantly upregulated miRNAs were found to be concurrently expressed in plasma. It was also found that miR-485-3p and miR-4433a-5p are potential markers for PTC in clinical diagnosis. Moreover, miR-204-3p and miR-4306 were confirmed as clinical biomarkers for PTC 71. Serum exosomal long noncoding RNA lnc-GNAQ-6:1 is lowly expressed in gastric cancer patients. The difference in expression levels might be used as a new diagnostic marker for disease assessment in large-scale gastric cancer studies 72. In addition, circular RNAs (circRNAs) are associated with cancer progression and development. In Cholangiocarcinoma (CCA), circ-0000284 (circHIPK3) was found to elevate lymphocyte antigen 6E (LY6E) expression by competitively binding miR-637Owing to circRNAs that are enriched and stable in exosomes. Exosome-mediated circ-0000284 has been reported to contribute to the development of cancer in surrounding normal cells 73. Exosomal circRNAs are easy to be detected due to stable structure, extensive expression, conserved sequence, and abundant content, thus providing additional evidence for conventional diagnostic methods and holding great promise as diagnostic markers for cancer.

### DNA

Exosomes are secreted by both viable and dying tumor cells and exosomal DNA (ExoDNA) is likely to be one of the most stable cargos due to protection by lipid bilayer 74. The discovery that exosomes contain tumor specific DNA mutations reinvented the clinical diagnosis of tumor mutations and opened up the possibility to perform liquid biopsy on patients. Exosomal DNA represents the entire genome and reflects the status of mutations in parental tumor cells 75. In non-small cell lung cancer (NSCLC), it has been shown that the mutant EGFR gene is enveloped in exosomes. Exosomal circulating mutations are present during early tumor development. About 50% of malignant melanomas have a BRAF (V600E) mutation, while ExoDNA mutant alleles have been reported in mutant cell lines. In pancreatic ductal adenocarcinoma (PDAC), KRAS mutations in ExoDNA were detected in 43.6% of early PDAC patients and in 20% of healthy controls 76. The packaged amount of exoDNA varies depending on the cancer type.^77^ The DNA content may be related to the size exosomal vesicles. High molecular weight tumor DNA fragments are specifically exported to exosomes. A comparative study by vagner et al found that larger vesicles (> 1 µm) contain more DNA and preferentially export genetic aberrations than smaller vesicles 78. Mutations KRAS and P53 were detected in exogenous DNA from pancreatic cancer cell lines and in the genomic DNA from the serum of pancreatic cancer patients 79. It has been demonstrated that the majority of DNA in exosomes derived from tumors is double stranded. Williams et al. definitively confirmed that dsDNA was present in tumor-associated exosomes 80. Compared with serum exo-DNA and tumor tissue DNA in patients with pheochromocytoma (PCC) and paraganglioma (PGL), dsDNA fragments are highly concordant with paired oncogenome. The definitive evidence of the presence of exosomal double-stranded DNA (dsDNA) shows, for the first time, that it can be used as a non-invasive genetic marker for the diagnosis and preoperative evaluation of PCCs and PGLs 44. Vaidya *et al.* performed a detailed analysis of the association between the NANOG gene family and exosomes 81. Differential NANOGP8 sequences from other embryonic stemness genes (OCT3/4, SOX2, etc.) may establish a set of exosomal-based diagnostic markers. Exosomes contain large dsDNA fragments spanning multiple genomic regions and harbor EGFR (T790M and L858R) 82 and BRAF (V600E) 83 gene mutations, proposing the possibility of tracing these mutations in plasma exosomes of NSCLC and/or melanoma patients through liquid biopsy. Exo-DNA has a short half-life and can accurately reflect tumor status in real time. In addition, exoDNA detection has potential clinical application in minimal residual disease and early warning of recurrence, contributing to better management of cancer and benefiting more patients.

### Proteins

Studies have shown the role for exosomal proteins in clinical diagnosis. Exosomes contain various tumor-associated proteins that reflect tumor statuses. In addition, they also contain proteins from cells. The membrane structure of exosomes protects proteins from external proteases and other enzymes 84,85. Due to their small sizes, strong permeability and high sensitivity, exosomal proteins from cancer cells can be used as biomarkers for cancer monitoring and efficacy evaluation, thereby helping in cancer diagnosis and early detection 86. Exosomal extracellular matrix protein 1 (ECM1) 87 and Alpha-2-HS-glycoprotein (AHSG) 88 respectively promote cancer progression and invasion in most tumors. A study using proteomic profiles confirmed that the combination of AHSG, ECM1, and carcinoembryonic antigen (CAE) improve the diagnostic potential of NSCLC 89. Moreover, melanoma secreted exosomes were found to contain intact tumor antigens to activate CD8+ T cells, and showed anti-cancer activities after being absorbed by dendritic cells, suggesting that CD63 in plasma exosomes can be used as a protein marker for melanoma 90. Exosomal membranes are homologous to cell membranes, and about 40% of cancer cell surface markers are found on the surface of exosomes. As biomarkers, exosomal proteins can provide a rich, stable, sensitive, and specific biological information. Kristine et al. 91 developed an EV array that coupled 37 antibodies targeting lung cancer-related proteins and CD9, CD63, and CD81 antibody groups to explore circulating exosomes from healthy subjects and patients with lung cancer. Proteomic characteristics of salivary and serum exosomes from lung cancer patients and healthy controls have been identified and comprehensively compared by liquid chromatography-tandem mass spectrometry (LC-MS/MS) 92. Certain special proteins are only present on the exosomes derived from malignant cells 93. Compared to healthy controls, plasma levels of epithelial cell adhesion molecule (EpCAM) -positive exosomes have been found to be significantly higher in breast cancer patients 94. Protein phosphorylation is the fundamental, most prevalent mechanism controlling diverse cellular physiological functions, and few phosphoproteins have been identified as potential biomarkers to distinguish disease from health 95. A recent study showed that phosphoproteins in plasma exosomes are highly feasible as disease biomarkers. Using label free quantitative Phosphoproteomics, researchers identified 144 phosphoproteins in plasma EVs that were significantly higher in patients diagnosed with breast cancer compared to healthy controls. Glypican-1 (GPC-1), the cell surface proteoglycan 96, is exclusively present on exosomes from malignant cells and is overexpressed in breast and pancreatic cancers. Application of GPC-1 as a diagnostic marker for colorectal cancer (CRC) has also been reported 97. Gammaglutamyltransferase 1 (GGT1), a cell surface enzyme, and CD9 in exosomes are present in PCa patients 98. In conclusion, even though there are associated difficulties and problems in the study of exosomal proteomics, it provided a novel method for cancer diagnosis 99.

### Glycoconjugates

Studies on exosomes have addressed the role of glycosylation. The surface of exosomes consists of a polysaccharide canopy attached to surface proteins and certain outer leaflet lipids. Malignancy is best characterized by the feature of aberrant sialylation in glycoproteins and glycolipids, which have been implicated in cancer progression 100. It has been established that plasma membrane‐associated ganglioside sialidase (NEU3) is up-regulated in a variety of cancers, including colon and prostate cancers. Keiko Hata *et al.* found different levels of ganglioside activity in cancer patients, and evaluated the sialidase NEU2 subtype as a potential biomarker for human cancer diagnosis by enzyme-linked immunosorbent assay (ELISA) 54. TEXs are somewhat different because their glycan characteristics vary with the expression of various glycosidases and glycosyltransferases 101. For example, hyaluronic acid synthase 3 levels are highly expressed in some cancers, leading to long polymers that synthesize hyaluronic acid, promote cancer growth and the biosynthesis of exosomes. Cancers can express heparanase to cleave the heparin sulfate proteoglycan chain. However, addition of exogenous heparinase was shown to alleviate the apparent spatial block of SDC budding, and increases the release of exosomes containing SDC 102. In addition to special surface proteins and glycans, certain types of exosomal lipids have been studied. Glycolipids from exosomes have also been considered to be potential cancer biomarkers elsewhere. For example, glycosphingolipids such as hexosylceramides (HexCer) and lactosylceramides (LacCer) were abundantly enriched in PCa cells-derived exosomes 95. Among them, phosphatidylserine (PS) is a type of phospholipid that is anchored within the inner leaflet of the normal cell membrane but is externalized in malignant and apoptotic cells. Y. T. Kang et al. reported that Tumor-related exosomes can be isolated by the PS targeting method 103. PS is not only specific to cancer cells, but has also been shown to have a regulatory effect on immune cells *in vivo*. This method has been used to isolate exosomes from samples, and confirmed that exosomal PS expression is not only a characteristic of vesicle formation, but also a PS mediated immune response on the surface of exosomes from NK cells, which contribute to tumor diagnosis and treatment.

## Exosomal detection and monitoring technology

Although TEXs characteristics enhance their potential as clinical biomarkers, effective methods for isolation and detection have not been established. Differential ultracentrifugation (UC) has been a classical method for EV separation, at least, until recently 104. A variety of approaches for detecting exosomes have been established, which include the conventional nanoparticle tracking analysis (NTA) 105, ELISA 106, flow cytometry 107, digital detection 108, etc. The methods for exosomal quantification requires low sample bulk and high sensitivity. However, the step-by-step processing and analysis of exosomes is time and labor-consuming. Moreover, the samples are also vulnerable to external contamination 109. Faster and more directional extraction procedures such as alternating current (AC) chip and microfluidic chip have been developed. An AC microarray chip that can isolate exosomes and other nanoscale substances (EVs and cf-DNA) from the whole blood of pancreatic cancer patients within 20 minutes and perform *in situ* immunofluorescence analysis has been designed 48. This assay can distinguish between PDAC patients and healthy people, and can also detect patients with metastatic diseases. Xu and colleagues developed a two-stage microfluidic platform (ExoPCD-chip) that integrates on-chip separation and *in situ* electrochemical analysis of exosomes in serum 110. Zhou et al. designed a hairpin-like structure aptasensor that integrates the highly specific MUC1 aptamer with a hemin/G-quadruplex to detect exosomes in breast cancer 111. Compared to other methods, it was found to be a sensitive, simple and low-cost colorimetric method that could distinguish between breast cancer patients and healthy individuals. An aptasensor based on aptamer-capped Fe_3_O_4_ NPs was constructed for visual and label-free detection of PCa exosomes from plasma. Exosomes were directly isolated from clinical PCa plasma and detected by the aptasensor for the early diagnosis and staging of PCa. In addition to the new detection technology, there have been advances on the traditional detection methods, such as ELISA, to resolve reproducibility and sensitivity issues 112. Moreover, UC has also been combined with polymer precipitation to separate small extracellular vesicles in the serum of breast cancer patients 113. Lee et al. proposed a sandwich paper-based ELISA (p-ELISA) method 114 that is a portable diagnostic system with low cost, simple to operate and easy to use. It is efficient for the isolation/detection functions of targeted EVs/exosomes. The procedures for this method can be done within 2 h, and the SARBI p-ELISA assay provides strong binding due to the strong affinity between biotinylated Ab and streptavidin agarose. Exosome-containing microRNAs (exomiRs) are potential biomarkers for tumor diagnosis. A novel exosome-specific tumor diagnosis strategy was constructed by combining the rapid magnetic exosome-enrichment platform with the Ru(bpy)32+-polymer amplified electrochemiluminescence (ECL) strategy 115. To realize the rapid and efficient capture of TEXs, the researchers constructed a non-destructive detection mode of tumor liquid biopsy through the biological affinity identification platform of EpCAM antibody. The glypican-1 and CD63 biomarkers reflect the presence of PDAC, which was quantified within as little as 30 min by subsequent on-chip immunofluorescence analysis 48. Compared to healthy people, elevated glypican-1 in PDAC patients with 99% sensitivity and 82% specificity can improve early stage cancer diagnostics by ACE exosome capture and on-chip biomarker detection. Specificity and sensitivity are the two foremost concerns in biological detection 116. Yang et al. 117 measured the expression of miR-21 microRNA and TTF-1 mRNA in the exosomes containing EGFR or PD-L1 in human plasma using an immune biochip. Compared to the traditional immunomagnetic separation, immuno-biochip enhanced lung cancer diagnostic platform uses a shorter time and consumes less than 30 μL of samples. However, it is important to develop a variety of sensitive methods, including probes and biomaterials to isolate exosomes.

## Conclusion

The complexity of exosomes bring many challenges to isolate large pure and specific exosomes from mixture. Maybe, only a small subtype of exosomes in an abundance mixture has the feature of biomarkers. It is an important process to determine the specific or characteristic exosomes from the abundance mixture. The concentration of TEXs were an important parameter that indicate the tumor pathological circumstances. This means that the TEXs is more abundant in serum, which is helpful for cancer diagnosis.

Compared with conventional biopsy or liquid biopsy, exosome-based diagnosis has higher sensitivity and specificity. By combining exosome DNA and RNA sequencing tools, exosome proteomics analysis and immunoassay technology, it is expected that exosomes will gain widespread use in the diagnosis and treatment of cancer.

## Figures and Tables

**Table 1:** Exosomal cargos as biomarkers for various cancers

Cargos	Cancer type	Source	Method	Detection	Reference
**Nucleic acid**					
**RNA**					
**IncRNAs**					
lncRNA MIAT	GC	Serum	qRT-PCR detected serum exosomal MIAT	Compared to healthy controls, pre-treatment and recurrence, MIAT levels were higher in GC patients	21
IncRNA-ATB	HCC	Serum/plasma	NcRNAs were isolated from exosomes using the miRNeasy serum/plasma micro kit, characterized and analyzed.	lncRNA-ATB were correlated with TNM stage. The more tumor volume and CRP are, the more level of IncRNA-ATB is.	22
MALAT-1	NSCLC	Serum	To measure the expression levels of MALAT-1 contained in exosomes	Exosomal MALAT-1 was highly expressed in NSCLC patients	23
IncRNA-p21	PCa	Urine	Quantification of IncRNA molecules was performed by real-time PCR	IncRNA-p21 levels were elevated in PCa patients than BPH	24
HOTAIR	GBM	Serum/tumors	HOTAIR expression was measured using qRT-PCR.	HOTAIR levels from GBM patients were significantly higher than in the controls	25
miR-21, HOTAIR	LSCC	Serum	RT-PCR was performed to detect the expression levels of miR-21 and HOTAIR	The expressions of miR-21 and HOTAIR were significantly higher in patients with LSCC than vocal cord polyps	26
HOTAIR/MALAT1/MEG3	Cervical cancer	Cervicovaginal lavage specimens	Exosomal lncRNAs HOTAIR, MALAT1, and MEG3 were quantified by qRT-PCR.	Expression of HOTAIR, MALAT1 and MEG3 was predominantly observed in cervical cancer	27
H19	BC	Serum	To measure the levels of lncRNA H19 by using quantitative real-time PCR	H19 expression levels were upregulated in BC patients	28
**miRNAs**					
miR-20a/miR-21	cHL	Serum	Analysis of immunovirological data	miR-21 and miR-20a levels were highly associated with adverse clinical outcomes	29
miR-21/miR-27a/miR-375	BC	Serum	Clinical samples were used to test exosomal miRNAs by using the biosensor	miR-21, miR-27a and miR-375 levels were highly expressed in breast cancer cells	30
miR-3940-5p/miR-8069	PDAC	Urine	Novel biomarker candidates for PDCA were found using expression profiling, and validated in samples using 3D digital PCR	miR-3940-5p/miR-8069 ratio in urine exosomes was elevated in PDAC patients	31
miR-5189-3p	PTC	Plasma	Analysis of miRNA expression in PTC and NG	miR-5189-3p levels were significantly high in PTC patients	32
miR139/miR136/miR19/miR210	Bladder cancer	Urine	Detection of miRNAs using Real-time PCR and ELISA	miR139/miR136/miR19/miR210 were upregulated in cancer patients	33
let-7b-5p/miR-122-5p/miR-146b-5p/miR-210-3p/miR-215-5p	BC	Plasma	Exiqon miRNA qPCR panel was used to select candidate miRNAs, which were further analyzed using qRT-PCR	miR-122-5p was upregulated in the plasma of BC patients. Let-7b-5p was contrarily down-regulated in BC tissue	34
miR-142-3p/miR-142-5p/miR-223-3p	PCa	Semen	An altered miRNA expression pattern was revealed by a high throughput profiling analysis in PCa ,and validated miRNA	Exosomal miRNA-based molecular biomarkers have the potential to improve PCa diagnosis	35
miR-141-3p/miR-375	LARC	Plasma	Expression of exosomal miRNAs was profiled using a miRCURY LNA miRNA microarray	Exosomal miR-141-3p and miR-375 levels were higher in patients	36
miR-7977	LUAD	Serum	Exosomal miRNAs expression levels in serum were further validated by the qRT-polymerase chain reaction.	Inhibition of miR-7977 enhanced the proliferation, invasion, and suppressed apoptosis in A549 cells	37
**CtRNAs**					
hsa-circ-0004771	CRC	Plasma	The expression and diagnostic utility of circRNA were tested by qRT-PCR and ROC analysis	Expression of hsa-circ-0004771 in the serum of CRC patients was elevated	38
hsa-circ-0043603/hsa-circ-0062459	ESCC	Plasma and cell culture	The expression of profile was identifiedmicroarray and displayed by qRT-PCR.	hsa-circ-0062459 was used as a diagnostic biomarker	39
Circ-IARS	PCa	Plasma	qRT-PCR was used to measure the expression levels of circ-IARS	circ-IARS expression was up-regulated in pancreatic cancer tissues and in plasma exosomes of patients with metastatic disease	40
F-circEA	NSCLC	Peripheral blood	The presence of the EML4-ALK fusion gene in H2228 cells was verified by sequencing RT-PCR	F-circEA is specifically present in the plasma of EML4-ALK positive NSCLC patients	41
Circ SATB2	NSCLC	Serum	The expression of genes and proteins was detected by qPCR and western blot	circSATB2 was highly expressed in serum from lung cancer patients and was associated with lung cancer metastasis	42
**DNA**					
SOX2 DNA	GBM	Blood	To detect cancer-specific SNPs in exosomal SOX2 DNA sequence	SOX2 is stemness gene highly expressed in cancer stem cells	43
dsDNA	PCC/PGL	Serum	Compared the serum exosomal DNA and tumor tissue DNA from patients or mice with PCC or PGL	Serum-derived exosomal dsDNA in PCC and PGL was highly consistent with the paired tumor genome	44
**mRNAs**					
hnRNPH1	HCC	Serum	hnRNPH1 mRNA expression levels were measured using the TaqMan real-time PCR	Serum exosomal hnRNPH1 mRNA levels in HCC patients were significantly higher than in the other groups	45
hTERT	'Pan- cancer'	Serum	The expression of exosomal MT1-MMP mRNA in GC patients was detected using RT-PCR.	MT1-MMP mRNA expression levels were found to be higher in GC patients	46
MT1-MMP	GC	Serum	The expression of exosomal MT1-MMP mRNA in GC patients was detected using RT-PCR	MT1-MMP mRNA expression levels were found to be higher in GC patients	47
**Proteins**					
glypican-1/ CD63	PDAC	Whole blood/plasma/serum	On-chip immunofluorescence analysis permits specific identification and quantification of target biomarkers	The elevated glypican-1 was observed in metastatic but not in non-metastatic disease	48
HSP70	Solid cancers	Blood/urine	HSP70 concentration will be determine	HSP70-exosomes were detected and quantified in blood from patients with solid cancers.	49
GPC1	PCa	Serum	Python-based standardized data processing method was established to analyze exosomal GPC1 expression.	The abundance of GPC-1 was elevated in patients with pancreatic cancer.	50
cyclophilin A	NPC	Serum	Protein expression levels were validated by reverse transcription -quantitative polymerase chain reaction, Western blotting, and ELISA	CYPA levels of NPC patients were significantly higher than in normal cases	51
CD82	BC	Breast cancer tissue	Test CD82 expression in tissues and CD82 content in exosomes	CD82 expression levels in breast cancer tissues were significantly lower than in healthy and benign breast cancer tissues.	52
CPNE3	CRC	Plasma	CPNE3 expression levels were determined by RT-PCR, western blot, and immunohistochemistry	CPNE3 expression was elevated in CRC tissues.	53
**Glycoconjugates**					
Sialidases	PCC/PCa	Plasma	Serum sialidase was assessed by a sandwich ELISA method using two anti-NEU3 antibodies.	NEU3 is markedly upregulated in cancers	54
MUC1	Lung cancer	Plasma	LC-MS/MS by data-independent analysis mode identified proteins	MUC1 levels in NSCLC patients were 1.5-fold higher than in controls.	55
Glycoproteins	BC	Serum/plasma	Glycoproteins in extracellular vesicles (EVs) were identified through integrating quantitative glycoproteomics	20 glycoproteins were verified to be significantly higher in individual breast cancer patients.	56
**Lipids**					
PS	BC	Plasma	Highly specific and sensitive ELISA for the capture of PS-expressing tumor exosomes in blood from tumor mice	PS-expressing tumor exosome levels were significantly high in blood	57
Cholesterol	PCa	Urine	A high-throughput mass spectrometry quantitative lipidomic analysis was used to reveal the lipid composition of urinary exosomes in PCa patients	The highest significance was shown for phosphatidylserine and lactosylceramide	58

## Abbreviations

TEXs: tumor-derived exosomes
EVs: extracellular vesicles
GPC1: glypican 1
MVBs: multivesicular bodies
ILVs: intraluminal vesicles
ncRNA: non-coding small RNA
mRNAs: messenger RNAs
miRNAs: microRNAs
MCH I and II: major histocompatibility complexes I and II
HSP70,90: heat shock protein
lncRNAs: long non-coding RNAs
GC: gastric cancer
qRT-PCR: Quantitative reverse transcription-polymerase chain reaction
lncRNA-ATB: lncRNA-activated by tumor growth factor-beta (TGF-β)
HCC: hepatocellular carcinoma
MALAT-1: metastasis-associated lung adenocarcinoma transcript 1
PCa: prostate cancer
BPH: benign prostatic hyperplasia
GBM: Glioblastoma multiforme
LSCC: laryngeal squamous cell carcinoma
HOTAIR: Hox transcript antisense intergenic RNA
MEG3: maternally expressed gene 3
BC: breast cancer
cHL: classical Hodgkin lymphoma
NG: nodular goiter
LARC: locally advanced rectal cancer
miRNA: microRNA
LUAD: lung adenocarcinoma
CRC: colorectal cancer
circRNAs: circular RNAs
ESCC: Esophageal squamous cell cancer
EML4-ALK: Echinoderm Microtubule-associated protein-Like 4- Anaplastic Lymphoma Kinase
f-circM9: MLL/AF9 fusion gene
SOX2: Sex-determining region Y (SRY)-box 2
dsDNA: double-stranded DNA
PCC: Pheochromocytoma
PGL: paraganglioma
hnRNPH1: heterogeneous nuclear ribonucleoprotein H1
hTERT: human telomerase reverse transcriptase
MT1-MMP: Membrane Type 1-Matrix Metalloproteinase
NPC: nasopharyngeal carcinoma
CYPA: Cyclophilin A
CD82: cluster of differentiation 82
CPNE3: clinical significance of exosomal Copine III
CTCs: circulating tumor cells
CHB: chronic hepatitis B
LC: liver cirrhosis
DCIS: ductal carcinoma *in situ*
IDC: invasive ductal carcinoma
PTC: Papillary thyroid cancer
CCA: Cholangiocarcinoma
LY6E: lymphocyte antigen 6E
NSCLC: non-small cell lung cancer
PDAC: pancreatic ductal adenocarcinoma
ECM1: exosomal extracellular matrix protein 1
AHSG: Alpha-2-HS-glycoprotein
LC-MS/MS: liquid chromatography-tandem mass spectrometry
EpCAM: epithelial cell adhesion molecule
CAE: carcinoembryonic antigen
GPC1: glypican-1
CRC: colorectal cancer
GGT1: gammaglutamyltransferase 1
NEU3: plasma membrane-associated ganglioside sialidase
ELISA: enzyme-linked immunosorbent assay
HexCer: hexosylceramides
LacCar: lactosylceramides
PS: phosphatidylserine
UC: ultracentrifugation
NTA: nanoparticle tracking analysis
AC: alternating current
p-ELISA: paper-based ELISA
exomiRs: Exosome-containing microRNAs
ECL: electrochemiluminescence
MUC1: mucin 1
